# Moesin is an effector of tau-induced actin overstabilization, cell cycle activation, and neurotoxicity in Alzheimer’s disease

**DOI:** 10.1016/j.isci.2023.106152

**Published:** 2023-02-08

**Authors:** Adrian Beckmann, Paulino Ramirez, Maria Gamez, Elias Gonzalez, Jasmine De Mange, Kevin F. Bieniek, William J. Ray, Bess Frost

**Affiliations:** 1 Sam and Ann Barshop Institute for Longevity and Aging Studies, San Antonio, TX, USA; 2 Glenn Biggs Institute for Alzheimer’s and Neurodegenerative Diseases, San Antonio, TX, USA; 3 Department of Cell Systems and Anatomy, San Antonio, TX, USA; 4 University of Texas Health San Antonio, San Antonio, TX, USA; 5 The Neurodegeneration Consortium, Therapeutics Discovery Division, University of Texas MD Anderson Cancer Center, Houston, TX, USA

**Keywords:** Pathophysiology, Cellular physiology, Gene network

## Abstract

In Alzheimer’s disease, neurons acquire phenotypes that are also present in various cancers, including aberrant activation of the cell cycle. Unlike cancer, cell cycle activation in post-mitotic neurons is sufficient to induce cell death. Multiple lines of evidence suggest that abortive cell cycle activation is a consequence of pathogenic forms of tau, a protein that drives neurodegeneration in Alzheimer’s disease and related “tauopathies.” Here we combine network analyses of human Alzheimer’s disease and mouse models of Alzheimer’s disease and primary tauopathy with studies in *Drosophila* to discover that pathogenic forms of tau drive cell cycle activation by disrupting a cellular program involved in cancer and the epithelial-mesenchymal transition (EMT). Moesin, an EMT driver, is elevated in cells harboring disease-associated phosphotau, over-stabilized actin, and ectopic cell cycle activation. We further find that genetic manipulation of *Moesin* mediates tau-induced neurodegeneration. Taken together, our study identifies novel parallels between tauopathy and cancer.

## Highlights

• Network analyses reveal hub gene Moesin in a cancer-related module in tauopathy
• Tau-induced Moesin elevation drives actin stabilization and cell cycle activation
• Epithelial-mesenchymal transition features are present in tau transgenic *Drosophila*
• Tau-induced Moesin elevation may disrupt neuronal identity

## Graphical abstract

**Figure:**
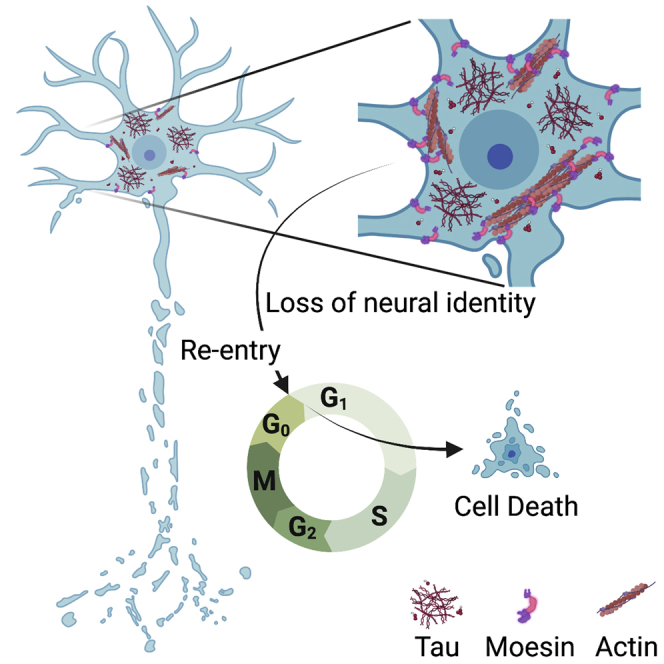

## Introduction

A neuropathological diagnosis of Alzheimer’s disease requires the presence amyloid β plaques and neurofibrillary tau tangles. Analyses of human brains have identified many additional cellular phenotypes of Alzheimer’s disease beyond amyloid β plaques and tau tangles, including upregulation of cell cycle-related proteins in terminally differentiated neurons.^1^^,^^2^ Post-mitotic cells such as neurons require persistently active cellular controls to maintain a quiescent, non-cycling state of terminal differentiation.^3^^,^^4^^,^^5^ Unlike cancer, in which uncontrolled cell division causes tumor formation, cell cycle activation in post-mitotic neurons is “abortive” in that it causes neuronal death rather than neuronal division.^6^^,^^7^^,^^8^^,^^9^ Mechanistically, multiple lines of evidence suggest that pathogenic forms of tau drive abortive cell cycle activation through over-stabilization of the actin cytoskeleton while simultaneously causing microtubule depolymerization.^10^^,^^11^^,^^12^^,^^13^^,^^14^^,^^15^ Deposition of tau-containing filamentous actin rods can be visualized as “Hirano bodies” in postmortem brains of patients with Alzheimer’s disease.^16^

Tau deposition follows a well-defined pattern in Alzheimer’s disease that permits differentiation of disease stages, termed “Braak staging.^17^” Tau-based positron emission tomography imaging of living individuals with Alzheimer’s disease indicates that tau deposition predicts areas of the brain that will degenerate over the following two years^18^ and that Braak tangle stage, but not amyloid stage, predicts age of onset and final Mini-Mental State Examination score.^19^^,^^20^ The association between dominantly inherited mutations in the gene encoding tau protein, *MAPT*, and frontotemporal dementias further demonstrates that tau dysfunction is sufficient to drive neurodegeneration in humans.^21^^,^^22^^,^^23^

In the current study, we sought to identify tau-induced drivers of actin stabilization and consequent abortive neuronal cell cycle activation in an effort to identify therapeutic targets for Alzheimer’s disease and related tauopathies. We applied a multi-system approach involving studies in postmortem brain tissue from patients with Alzheimer’s disease compared to mouse models of tau- and amyloid precursor protein (APP)-associated neurotoxicity across disease stage, followed by mechanistic studies in a *Drosophila* model of tauopathy. We identify *Moesin*, which is well known for its role in cancer metastasis and epithelial-mesenchymal transition (EMT),^13^^,^^24^ as a highly connected “hub” gene in network analyses from human Alzheimer’s disease and a mouse model of tauopathy. Turning to *Drosophila* for mechanistic studies, we find that Moesin elevation is co-incident with a disease-associated tau phosphoepitope, actin over-stabilization, and cell cycle activation in brains of adult tau transgenic flies. In line with our analyses in human Alzheimer’s disease and the known involvement of Moesin in the EMT, we find that expression of human transgenic tau causes a depletion of adhesion proteins associated with EMT as well as neuronal cellular adhesion proteins in the adult *Drosophila* brain. Genetic manipulation of Moesin mediates tau-induced actin over-stabilization, cell cycle activation, and neurodegeneration in brains of tau transgenic *Drosophila,* establishing that tau-induced elevation of Moesin is a causal factor driving neurotoxicity. Overall, our findings identify Moesin as a mechanistic link between pathogenic forms of tau, actin over-stabilization, and consequent abortive activation of the cell cycle.

## Results

### Network analysis of postmortem human Alzheimer’s disease brains identifies a large co-expression module related to cancer and the cytoskeleton

**Figure 1:**
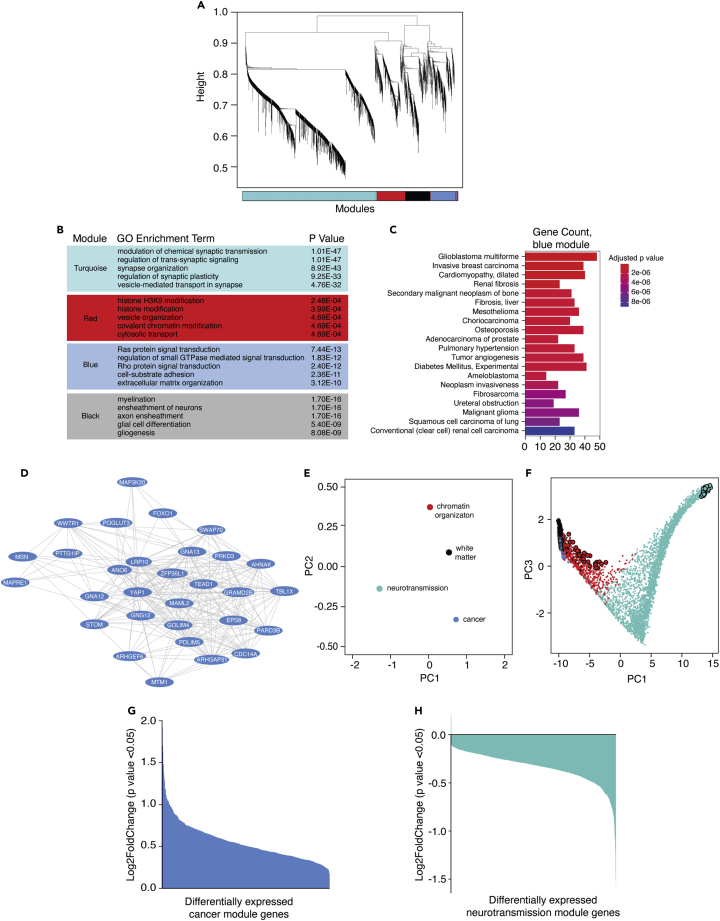
WGCNA of human Alzheimer’s disease brains and controls reveals a large co-expression network associated with cancer (A) Cluster dendrogram showing module assignment in human network analysis. Each vertical line in the clustering tree corresponds to a gene. Branches of the dendrogram group highly co-expressed genes and are used to identify modules based on hierarchical clustering. (B) Biological processes with the highest degree of significant enrichment based on Gene Ontology. The full tables of enriched processes are provided in Table S3. (C) Gene-disease association of the blue module. Bar plot depicts the top 20 most significant DisGeNET terms identified on the y axis and the number of genes populated in each term on the x axis. The full tables of all DisGeNET terms for each module are provided in Table S4. (D) Hub genes of the blue module. Each oval represents a node while each line represents the weighted connection between nodes. (E) Multidimensional scaling plot of the first and second principal components for module eigengenes identified by WGCNA. (F) Multidimensional scaling plot of the entire network using principal component three as a function of principal component one. Each point is a single gene. Larger points represent hub genes. Transcript levels of differentially expressed genes of the blue (G) and turquoise (H) modules. Bar plots show the log2FoldChange of patients with Alzheimer’s disease relative to control for each differentially expressed genes from the blue and turquoise modules. The full tables of all differentially expressed genes for each module are provided in Table S2. Colors within each figure correspond to the module assignment for each group of co-expressed genes.

Two major limitations of traditional RNA sequencing (RNA-seq)-based differential gene expression analysis are the inability to 1) understand the relationships between expressed genes and 2) stratify genes in a biologically meaningful manner. Weighted gene co-expression network analysis (WGCNA) presents an advantage over differential gene expression analysis by using RNA-seq data to analyze relationships between co-expressed genes, cluster groups of highly co-expressed genes into modules, and identify “hub genes” within each co-expression module.^25^ To gain greater insight into the transcriptional networks that govern cytoskeletal stabilization and cell cycle activation in Alzheimer’s disease, we performed WGCNA using publicly available RNA-seq data from temporal cortex of postmortem human control (n = 57) and Alzheimer’s disease (n = 82) patients generated by the Accelerating Medicines Partnership – Alzheimer’s Disease (AMP-AD) (Table S1). Based on WGCNA, we identify four distinct groups, or “modules,” of highly co-expressed genes within the human dataset (Figure 1A and Table S2).

To identify which, if any, of the co-expression modules were related to the cell cycle and/or cytoskeletal organization, we performed biological enrichment analysis using Gene Ontology (GO).^26^^,^^27^ We find that the blue module, composed of 600 genes, is significantly associated with cellular processes, including Ras and Rho signal transduction, that are involved in cancer (Figure 1B and Table S3). In addition, this module is associated with GO terms related to actin dynamics including actin filament assembly and actin bundling (Table S3). DisGeNET^28^ analysis of each module reveals that the blue module is indeed associated with various malignancies (Figure 1C and Table S4). Based on the link between cancer and cell cycle dysregulation, we selected the blue, cancer-related module for deeper investigation into potential drivers of cell cycle activation in Alzheimer’s disease.

“Hub genes” are defined as the most highly connected genes within a module. We identified hub genes of each module by ranking genes according to their intramodular connectivity (k_in_) and selecting the top 1-5% of the most highly connected genes (Figure 1D). Consistent with links between the blue module and cancer, we find that many blue module hub genes, such as *Moesin* (*MSN)*, *YAP1*, *TEAD1*, and *WWTR1* are well known for their role in cancer and mediate the EMT.^29^^,^^30^^,^^31^^,^^32^ Module eigengenes, defined as the first principal component of a module, can be used to measure the degree of similarity between modules in a network.^33^ Based on principal component analysis (PCA), we find a negative association between eigengenes of the blue cancer-related module versus eigengenes of the turquoise “neurotransmission” module (Figure 1E). Comparing the module eigengenes to principal component two reveals that the negative association between the blue cancer-related module and the turquoise neurotransmission module is driven primarily by their respective hub genes (Figure 1F). As further evidence of a negative association between the cancer and neurotransmission modules, we find that all differentially expressed genes of the cancer module are upregulated in brains of patients with Alzheimer’s disease (Figure 1G), while almost all differentially expressed genes of the neurotransmission module are downregulated in brains of patients with Alzheimer’s disease (Figure 1H). Taken together, our human network analyses suggest that human Alzheimer’s disease brains undergo upregulation of biological processes associated with cancer alongside downregulation of biological processes associated with neurotransmission and neuronal identity.

### Age-dependent network analysis of rTg4510 tau transgenic mice and J20 APP transgenic mice identifies biological processes in human Alzheimer’s disease that are driven by pathogenic tau and are conserved across disease stage

Limitations of a gene expression network constructed from late-stage postmortem human Alzheimer’s disease brain tissue are 1) the presence of co-pathologies, which do not allow one to differentiate between changes that are a specific consequence of pathological forms of tau, amyloid β, or other events such as vascular damage and 2) the inability to determine how co-expression networks change as the disease progresses. To determine the specific consequences of pathological tau versus amyloid β on gene expression networks and to identify changes that are conserved across disease stage, we performed WGCNA using RNA-seq data from tau transgenic rTg4510 mice and APP transgenic J20 mice.

**Figure 2:**
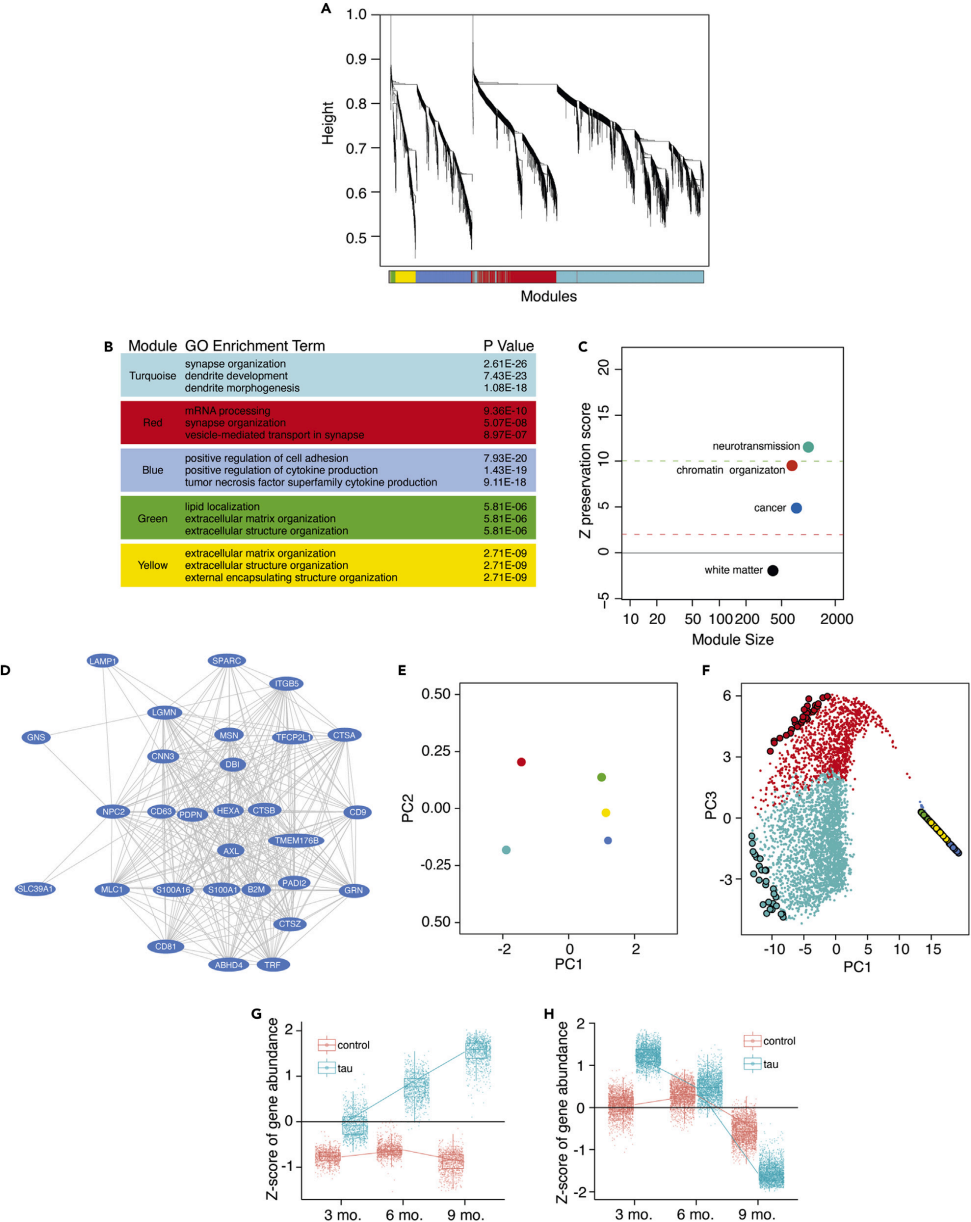
WGCNA of control and tau transgenic mice across aging reveals pathways in human Alzheimer’s disease that are driven by tau (A) Cluster dendrogram showing module assignment in tau transgenic network analysis. Vertical lines in the clustering tree each correspond to a gene. Branches of the dendrogram group highly co-expressed genes and are used to identify modules based on hierarchical clustering. (B) Table showing the three most significantly enriched terms for each module based on Gene Ontology. (C) Summary statistics for human module preservation in the tau transgenic network. The composite of all preservation statistics is calculated from module preservation including using summarized statistics from Zdensity and Zconnectivity-based statistics which are included in Figure S2. (D) Hub genes of the blue module. Each oval represents a node while each line represents the weighted connection between each node. (E) Multidimensional scaling plot of the first and second principal components for module eigengenes and identified by mouse WGCNA. (F) Multidimensional scaling plot of the entire mouse network using principal component three as a function of principal component one. Each point is a single gene. Larger points represent hub genes. Box and whisker plots show gene expression changes from the (G) blue and (H) turquoise modules at three, six, and nine months of age. Gene expression changes for other modules are included in Figure S4. The full table of all modules and the associated genes for each module across aging can be found in Table S8.

We first constructed a co-expression network using RNA-seq data from brains of three-, six-, and nine-month-old rTg4510 tau transgenic mice. This model features transgenic *CaMKIIa*-driven forebrain expression of the human *MAPT* gene carrying the disease-associated *P301L* mutation^34^^,^^35^ (referred to hereafter as “tau transgenic mice” for simplicity). Based on WGCNA, we identify five co-expression modules in brains of tau transgenic mice (Figure 2A). Similar to human Alzheimer’s disease, we find that the largest co-expression module, turquoise, is related to processes involved in neurotransmission and neuronal identity (Figure 2B). The blue, green, and yellow modules are closely related but form distinct modules. Based on GO analyses, these modules are associated with terms such as extracellular matrix organization, cellular adhesion, and immune response (Figure 2B and Table S5).

We next constructed a separate network using RNA-seq data from brains of J20 mice aged to six, eight, ten, and twelve months. The J20 mouse model transgenically expresses the human amyloid precursor protein (*APP*) gene harboring two disease-associated mutations (*APP*^*KM670/671NL*^ [Swedish]^36^ and *APP*^*V717F*^ [Indiana])^37^^,^^38^ driven by the platelet-derived growth factor (PDGF)-β promoter (referred to hereafter as “APP transgenic mice” for simplicity). As reported previously, we find that the APP network as whole lacks clear separation among modules and that modules do not change significantly across aging (Figures S1A–S1H, Tables S6 and S7).^39^ Together, these data suggest that modules in the APP network do not significantly change across disease stage and are transcriptionally similar to control mice.

We next asked whether the modular structure or “network signatures” of the human network are a consequence of pathogenic tau and/or Aβ. Module statistics of a reference network (human) can be used to quantify which aspects, termed “patterns of connectivity,” are preserved in a second test (mouse) network.^40^ Network statistics can be scored using a system in which Z-scores greater than ten are evidence of strong preservation, Z-scores between two and ten are evidence of weak to moderate preservation, and Z-scores below two are indicative of no module preservation. Patterns of connectivity among the neurotransmission, chromatin organization, and cancer modules in the human network are well preserved in the network generated from tau transgenic mice across aging (Figures 2C and S2). While tau transgenic mice have a generally greater degree of preservation than APP transgenic mice, we find that the neurotransmission and chromatin organization modules identified in the human network are well preserved in both the tau and APP transgenic networks (Figures S3A and S3B). The human cancer-related blue module is more strongly preserved in the tau transgenic mouse network than the APP mouse network (Figure S3A). Overall, these data suggest the “cancer” module identified in human network analysis is largely driven by pathogenic tau.

We next identified hub genes within each module of the mouse network (Figure 2D and Table S8). Within the tau transgenic network, we find that *Moesin* and other growth- and cell motility-related genes including *Lgmn*,^41^ *Pdpn*,^42^ and *Tfcp2l1* are hub genes within the blue module.^43^ Alongside the closely related yellow and green modules, we find that blue module is negatively correlated with the neurotransmission module based on PCA, similar to our findings in human Alzheimer’s disease (Figure 2E). Viewing the entire network and associated module hub genes along their principal components reveals that the negative association between the blue/yellow/green modules and the turquoise module is driven primarily by their respective hub genes (Figure 2F). Transcript levels of genes within the blue module are significantly elevated compared to control across time points; this difference becomes more pronounced with age (Figure 2G, similar to the green and yellow modules, Figure S4). Transcript levels of genes within the neurotransmission module are elevated in tau transgenic mice at three months but are significantly depleted in tau transgenic mice compared to control by nine months (Figure 2H). Taken together, our WGCNA and module preservation analyses identifies co-expression networks that are well preserved between human Alzheimer’s disease and tau transgenic mice, suggesting that these changes are a consequence of pathogenic tau.

### Moesin is elevated at the protein level in human Alzheimer’s disease and is co-incident with pathogenic tau, filamentous actin, and cell cycle activation in a *Drosophila* model of tauopathy

**Figure 3:**
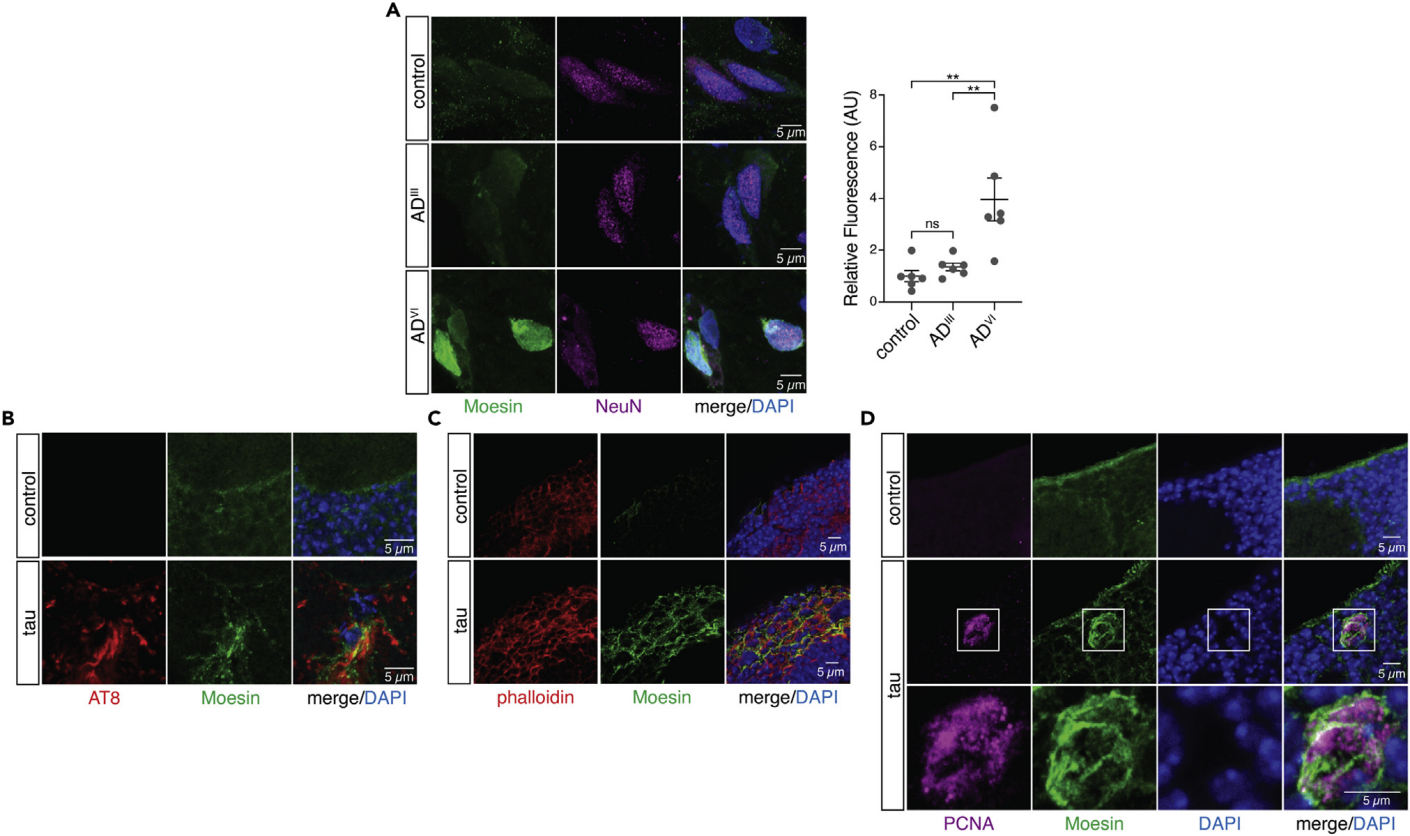
Moesin is elevated in human Alzheimer’s disease and is co-incident with disease-associated phosphotau, filamentous actin, and cell cycle activation in brains of tau transgenic *Drosophila* (A) Moesin is elevated in neurons of the frontal cortex in patients with Alzheimer’s disease at Braak V/VI based on immunofluorescence (one-way ANOVA, Tukey’s test). (B) Elevated levels of Moesin occur at sites where disease-associated phosphorylated forms of tau deposit in brains of tau transgenic *Drosophila*. (C) Moesin elevation is co-incident with filamentous actin enrichment in the medulla of tau transgenic *Drosophila*. (D) 100% of PCNA-positive cells and cell clusters colocalize with focal Moesin elevation in brains of tau transgenic *Drosophila*. All flies are ten days old. Values are mean ± SEM, n = 6 biologically independent replicates per genotype, ∗p < 0.05, ∗∗p < 5.0x10^−3^, ∗∗∗∗p < 5.0x10^−5^. Full genotypes are listed in Table S10.

We became interested in Moesin as a candidate mediator of actin over-stabilization and cell cycle activation based on its presence as a WGCNA hub gene in co-expression networks of both human Alzheimer’s disease and tau transgenic mice, its known role as a mediator of cancer and the EMT, and its ability to regulate actin. Ezrin, Radixin, and Moesin (ERM) proteins crosslink filamentous actin to the plasma membrane.^44^^,^^45^ Studies in breast cancer indicate that aberrant activation of Moesin causes over-stabilization of the actin cytoskeleton, which mediates EMT and metastasis.^46^^,^^47^ We first asked if Moesin is elevated at the protein level in postmortem brains of patients with early and late stages of Alzheimer’s disease compared to age-matched controls (Table S9). As predicted by WGCNA, we detect a significant elevation in Moesin protein levels in frontal cortex of postmortem human Alzheimer’s disease brains at Braak V/VI based on immunostaining (Figure 3A).

After validating that overall levels of Moesin protein are significantly elevated in postmortem brains of patients with Alzheimer’s disease, we turned to *Drosophila* for additional functional and mechanistic analyses of Moesin dysregulation in the adult brain. Panneuronal expression of human wild-type tau and disease-associated tau mutants in *Drosophila* recapitulate many aspects of Alzheimer’s disease and related primary tauopathies including progressive neurodegeneration,^48^ DNA damage,^49^ and synapse loss.^50^ In addition, neurons of tau transgenic *Drosophila* undergo an abortive cell cycle activation via a neurodegenerative process that shares many features of metastatic cancer cells and less differentiated cell types, including over-stabilization of filamentous actin,^11^^,^^51^ nuclear pleomorphism,^52^^,^^53^ loss of heterochromatin-mediated transcriptional silencing,^54^^,^^55^ and activation of transposable elements.^56^^,^^57^^,^^58^ As our co-expression analyses of human Alzheimer’s disease and tau transgenic mice indicated that Moesin involvement in tauopathy is not restricted to wild-type versus mutant tau, we analyzed Moesin in a *Drosophila* model of tauopathy that features panneuronal expression of a disease-associated mutant form of tau (tau^R406W^, referred to hereafter as “tau transgenic *Drosophila*” for simplicity)^48^ that features a moderate degree of neurotoxicity that is well suited for genetic analyses. All analyses were performed at day 10 of adulthood.

We performed immunofluorescence-based analysis of adult brains of tau transgenic *Drosophila* to visualize the relationship between Moesin and AT8, an antibody that detects disease-associated tau protein phosphorylated at serines 202 and 205.^59^ We observe focal elevation of Moesin at sites of AT8 enrichment (Figure 3B). Similarly, we find that Moesin is elevated at sites of filamentous actin stabilization based on phalloidin staining (Figure 3C) and sites of cell cycle activation based on co-labeling with an antibody that detects proliferating cell nuclear antigen (PCNA) (Figure 3D). As evidence of a tight link between Moesin and cell cycle activation, we observed presence of Moesin in every incidence of PCNA positivity in brains of tau transgenic *Drosophila*. Taken together, this series of experiments suggest that the Moesin elevation we observe in human Alzheimer’s disease is indeed a consequence of pathogenic forms of tau and correlates with actin over-stabilization and cell cycle activation.

### Brains of tau transgenic *Drosophila* exhibit canonical cellular hallmarks of EMT and depletion of neuronal adhesion proteins

Based on the known role of Moesin as a driver of EMT, we became interested in the potential involvement of an EMT-like pathway in tauopathy. During EMT, transdifferentiation of epithelial cells into mesenchymal cells is important for wound healing^60^^,^^61^ and organ development.^62^ Over the course of EMT, epithelial cells lose cellular adhesion proteins and acquire properties akin to mesenchymal stem cells including migratory capacity and multipotency.^63^ In addition to its physiological function, EMT can also drive disease. In breast cancer, for example, EMT disrupts the terminally differentiated epithelial phenotype to facilitate tumor metastasis,^64^^,^^65^ cell cycle activation, and consequent malignancy.^24^^,^^66^^,^^67^

**Figure 4:**
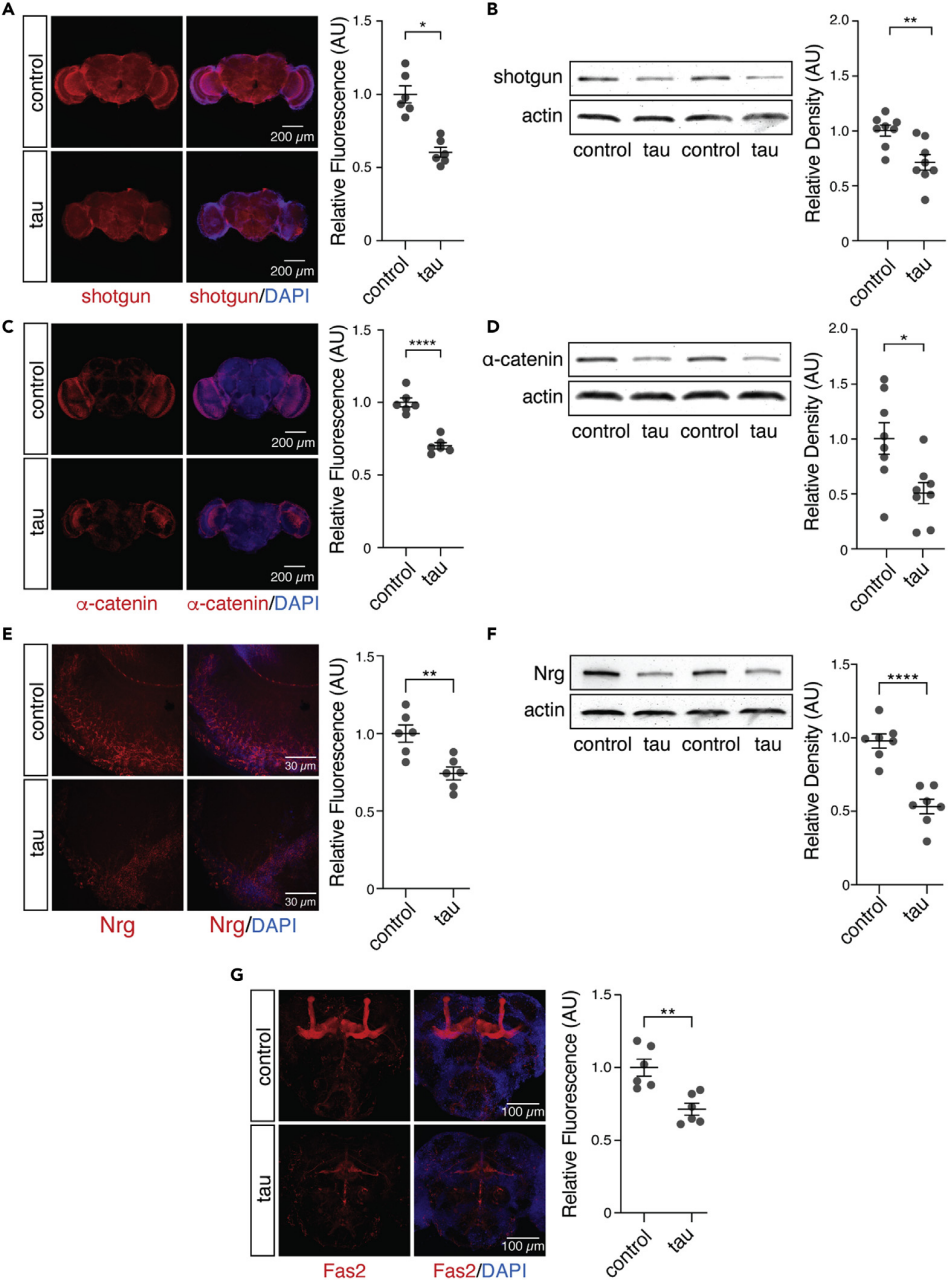
Hallmarks of EMT are conserved in brains of tau transgenic *Drosophila* (A–F) Protein levels of shotgun (A and B), α-catenin (C and D), and Neuroglian (E and F) are depleted in brains of tau transgenic *Drosophila* compared to control based on immunostaining and western blotting. (G) Fas2 is depleted in brains of tau transgenic *Drosophila* based on immunostaining. n = 6–8 biologically independent replicates per genotype. All flies were ten days old. Values are mean ± SEM Unpaired, two-tailed Student’s *t* test, ∗p < 0.05, ∗∗p < 0.005, ∗∗∗p < 5.0x10^−4^, ∗∗∗∗p < 5.0x10^−5^. Full genotypes are listed in Table S10.

During EMT, changes in the actin cytoskeleton cause downregulation of adhesion molecules such as cadherin 1^68^^,^^69^ and catenin alpha 1.^70^ We find that shotgun and α-catenin, the *Drosophila* homologs of human cadherin 1 and catenin alpha 1, are significantly decreased in brains of tau transgenic *Drosophila* compared to controls (Figures 4A–4D). We next investigated cellular adhesion proteins that are important for neuron-specific functions in *Drosophila*. We detect a significant reduction in Neuroglian (Nrg) (Figures 4E and 4F) and Fasciclin 2 (Fas2) (Figure 4G), which regulate synapse formation, axon pathfinding, and neurite extension,^71^^,^^72^^,^^73^ indicating that cellular adhesion proteins that are important for neuronal function are also depleted in tau transgenic *Drosophila.* Taken together, depletion of adhesion molecules that are canonical hallmarks of EMT alongside loss of neuronal adhesion proteins in brains of adult tau transgenic *Drosophila* is consistent with Moesin elevation and suggests that pathogenic tau drives neuronal changes that mimic cellular phenotypes that occur in EMT.

### Moesin activation causally mediates tau-induced actin over-stabilization, cell cycle activation, and neuronal death

**Figure 5:**
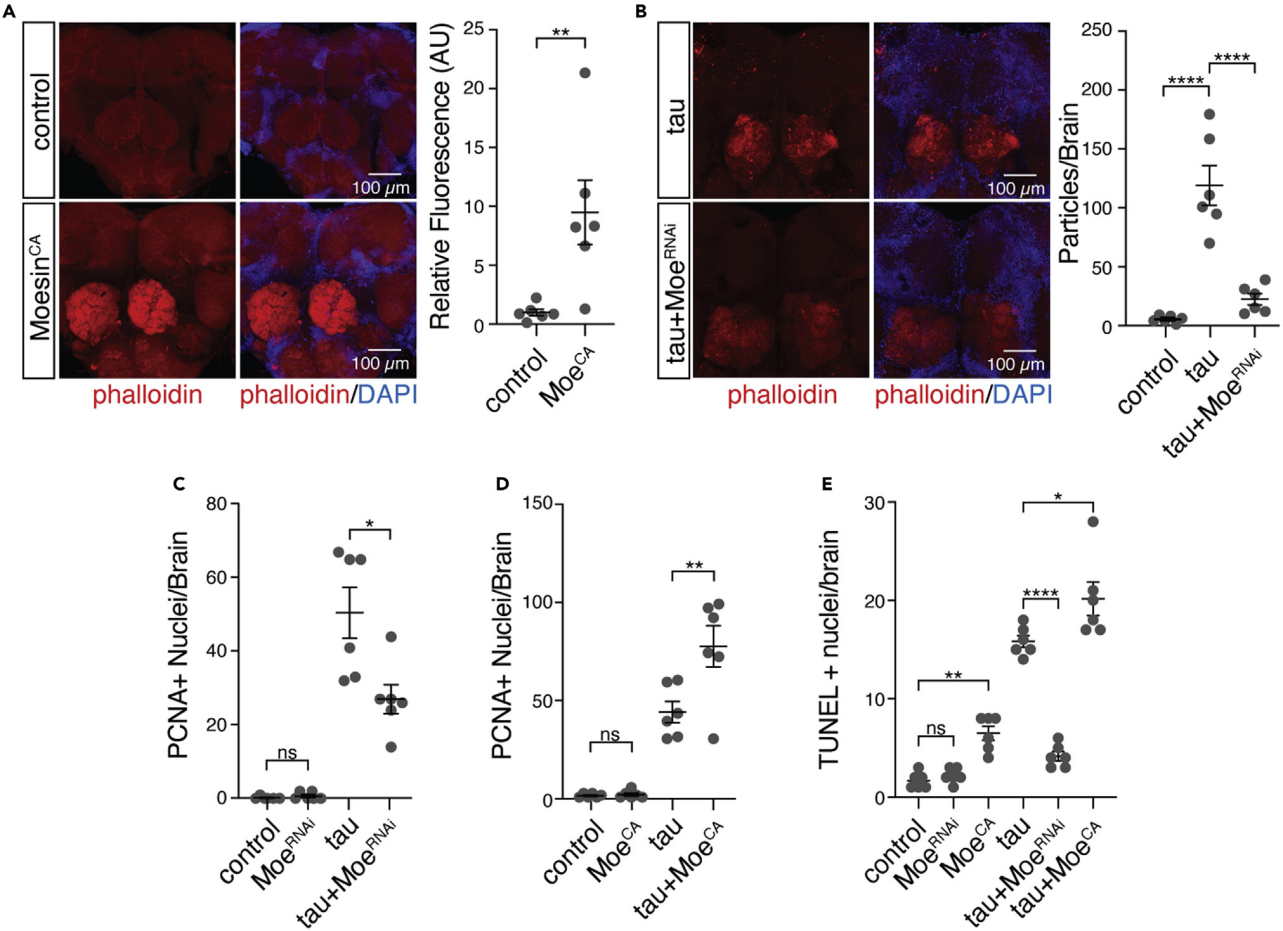
Moesin activation in tau transgenic *Drosophila* is causally connected to filamentous actin formation, cell cycle activation, and neuronal death (A) Filamentous actin is elevated in the central brain of *Drosophila* harboring a constitutively active *Moesin* mutant relative to control based on phalloidin staining (unpaired, two-tailed Student’s *t* test). (B) RNAi-mediated Moesin knockdown decreases levels of filamentous actin in brains of tau transgenic *Drosophila* based on phalloidin staining (one-way ANOVA, Tukey’s test). (C and D) (C) Moesin knockdown significantly suppresses tau-induced cell cycle activation while (D) constitutive activation of Moesin significantly enhances tau-induced cell cycle activation based on PCNA (one-way ANOVA, Tukey’s test). (E) Moesin knockdown significantly suppresses tau-induced neuronal death while constitutive activation of Moesin significantly enhances tau-induced neuronal death based on TUNEL staining (one-way ANOVA, Tukey’s test). All flies are ten days old. Values are mean ± SEM, n = 6 biologically independent replicates per genotype, ∗p < 0.05, ∗∗p < 5.0x10^−3^, ∗∗∗∗p < 5.0x10^−5^. Full genotypes are listed in Table S10.

We continued our studies in *Drosophila* to determine if tau-induced Moesin activation causally mediates actin over-stabilization, cell cycle activation, and neurotoxicity. We find that panneuronal overexpression of a constitutively active form of Moesin, Moesin^T559D^ (hereafter referred to as “Moesin^CA^”), is sufficient to significantly elevate levels of filamentous actin in the adult *Drosophila* brain based on phalloidin staining (Figure 5A). Conversely, panneuronal RNAi-mediated knockdown of Moesin in tau transgenic *Drosophila* significantly decreases levels of filamentous actin (Figure 5B) In addition, panneuronal RNAi-mediated knockdown of Moesin significantly suppresses, while overexpression of constitutively active Moesin significantly enhances, cell cycle activation in brains of tau transgenic *Drosophila* based on PCNA (Figures 5C, 5D, and S5A). Based on TUNEL, which detects DNA damage associated with apoptosis, we find that panneuronal RNAi-mediated knockdown of Moesin significantly suppresses neuronal death while panneuronal overexpression of Moesin significantly enhances neuronal death in tau transgenic *Drosophila* (Figures 5E and S5B). Collectively, these data suggest that pathogenic tau drives the elevation of Moesin detected in human Alzheimer’s disease and that aberrant Moesin activation mediates actin over-stabilization, cell cycle activation, and consequent neuronal death in tauopathy.

## Discussion

Since the initial discovery in 1996 linking pathogenic forms of tau to upregulation of the cell cycle-related protein p16 in neurons of the adult Alzheimer’s disease brain,^74^ a wealth of literature has implicated tau as a driver of abortive cell cycle activation in neurons.^75^^,^^76^^,^^77^^,^^78^ Work in multiple model systems has identified a series of cellular events connecting pathogenic forms of tau to cell cycle re-entry, including over-stabilization of the cytoskeleton,^79^^,^^80^ disruption of microtubule stability,^81^ disruption of nuclear architecture,^82^^,^^83^ loss of heterochromatin-mediated gene silencing,^84^^,^^85^ and activation transposable elements.^56^^,^^58^ In the current study, we sought to investigate the biological underpinnings of tau-induced cytoskeletal stabilization and consequent neuronal cell cycle activation.

In two separately constructed networks from postmortem brain tissue from patients with sporadic Alzheimer’s disease and tau transgenic mice at three stages of disease, we identified *Moesin* as a hub gene within an expression module associated with cancer and EMT. The modular structure of the human network, which involves deposition of human wild-type tau in brains of affected individuals, is well preserved in the network derived from tau transgenic mice despite transgenic overexpression of a disease-associated tau mutation in this model, indicating that the rTg4510 mouse model of tauopathy recapitulates changes that occur in sporadic human Alzheimer’s disease. In addition, these data suggest that co-expression networks in the human Alzheimer’s disease brain are not a simple consequence of neuronal loss as the mouse network consists of both control and tau transgenic mice at early-, mid-, and late-stage disease.

Moving into the *Drosophila* brain for mechanistic studies, we find a causal association between Moesin activation, filamentous actin formation, and cell cycle re-entry and that cellular hallmarks of EMT are present in *Drosophila* tauopathy. Interestingly, panneuronal activation of Moesin in the absence of pathogenic tau appears to differentially affect specific subsets of neurons as the largest qualitative elevations of filamentous actin were observed in the antennal lobes of the *Drosophila* brain. We also find that Moesin is elevated at the protein level in postmortem human brain from patients with Alzheimer’s disease, further suggesting a high degree of conservation between tau transgenic *Drosophila* and human Alzheimer’s disease.

Our findings implicating Moesin dysregulation in Alzheimer’s disease and related tauopathy converge with those of the National Institute on Aging’s Accelerating Medicines Partnership – Alzheimer’s Disease consortium, who have nominated Moesin as a drug target for Alzheimer’s disease (https://agora.ampadportal.org/genes/genes-router:gene-details/ENSG00000147065) based on genomic and proteomic data from human Alzheimer’s disease samples. Our identification of *Moesin* as a hub gene in human and mouse tauopathy networks aligns with the findings of the consortia, and our studies in *Drosophila* provide the mechanistic insight into the consequences of Moesin activation in tauopathy that are critical for drug development.

Cellular differentiation is the process of a cell changing from a multi- or pluripotent, less specialized cell into a specialized cell type. Some basic biological functions, such as EMT, require dynamic shifts between programs that maintain cellular identity and those that promote cellular plasticity. Multiple lines of evidence suggest that maintaining a terminally differentiated state is an active process that requires persistently active cellular controls.^86^ Mechanistically, the cytoskeletal remodeling that occurs with EMT causes breakdown of cell-to-cell connections and depletion of proteins that maintain an epithelial identity. In neurons, maintenance of cellular identity is an active process controlled by “terminal neuronal selector proteins,” key transcription factors that are in part regulated by the extracellular environment.^87^^,^^88^^,^^89^^,^^90^ Several nodes that we and others have identified within the cascade of tau-induced neurotoxicity (e.g. actin over-stabilization,^11^ nucleoplasmic reticulum expansion,^52^^,^^91^ heterochromatin relaxation and consequent expression of development-associated genes,^54^ cell cycle activation in neurons,^78^^,^^92^ and transposable element activation^56^^,^^58^) are present in less- developed cell types and in cells that have undergone de-differentiation.^93^^,^^94^^,^^95^^,^^96^^,^^97^ Indeed, induced neurons from patients with Alzheimer’s disease are reported to activate de-differentiation pathways.^98^ Based on our findings in the current study, as well as these parallels between cellular phenotypes in tauopathy and those of more immature cells, we speculate that pathogenic forms of tau drive neurodegeneration by disrupting the cellular program that is responsible for maintaining a terminally differentiated neuronal state.

### Limitations of the study

While our studies are guided by analyses in sporadic human Alzheimer’s disease brain, our subsequent analyses in mouse and *Drosophila* models of Alzheimer’s disease and related tauopathies rely on disease-associated mutations that model familial forms of Alzheimer’s disease or familial forms of frontotemporal dementia associated with *MAPT* mutation. Despite this limitation, we were struck by the high degree of preservation between transcriptional networks of tau transgenic mice and sporadic human Alzheimer’s disease, as well as conservation of Moesin elevation in tau transgenic *Drosophila*. In addition, while analyses in tau transgenic *Drosophila* reveal a tight co-incidence of Moesin elevation and cell cycle activation as detected by PCNA, future neuropathological analyses of human Alzheimer’s disease brain are required to determine if Moesin activation and aberrant cell cycle activation are tightly linked in the human condition. As several cell cycle-associated proteins that are elevated in human Alzheimer’s disease brain have additional cellular functions,^99^^,^^100^^,^^101^ use of multiple markers of cell cycle activation in human brain analyses would allow us to more confidently assess the link between Moesin and aberrant cell cycle activation in the human brain.

## STAR★Methods

### Key resources table

**Table:** 

REAGENT or RESOURCE	SOURCE	IDENTIFIER
**Antibodies**		

actin	Developmental Studies Hybridoma Bank	JLA 20
alpha-catenin	Developmental Studies Hybridoma Bank	DCAT-1
Fasciclin2	Developmental Studies Hybridoma Bank	1D4
Moesin	Dan Kiehart lab	NA
NeuN	Abcam	ab134014
Neuroglian	Developmental Studies Hybridoma Bank	BP 104
PCNA	Dako	M0879
phosphoTau (AT8)	Thermo Scientific	MN1020, RRID:AB_223647
shotgun	Developmental Studies Hybridoma Bank	DCAD2
β-tubulin	Abcam	ab179513
Alexa Fluor 488 (Mouse)	Invitrogen	A21042
Alexa Fluor 488 (Rabbit)	Invitrogen	A11034
Alexa Fluor 488 (Rat)	Invitrogen	A11006
Alexa Fluor 555 (Mouse)	Invitrogen	A21424
Alexa Fluor 555 (Rat)	Invitrogen	A21434
Alexa Fluor 647 (Mouse)	Invitrogen	A21235
Biotin Conjugated Mouse Secondary	Southern Biotech	1010-08

**Biological samples**		

Human brain tissue	Mayo Clinic, Dennis Dickson	NA

**Chemicals, peptides, and recombinant proteins**		

phalloidin	Cell Signaling Technologies	8953
DAPI	Southern Biotech	0100-20

**Critical commercial assays**		

FragEL DNA Fragmentation Detection Kit, Colorimetric (TUNEL)	Calbiochem	QIA33

**Deposited data**		

Human RNA sequencing data, control and Alzheimer’s disease samples	Accelerating Medicines Partnership - Alzheimer’s Disease	syn3163039
Mouse RNA sequencing data, control, rTg4510	Gene Expression Omnibus	GSE186140
Mouse RNA sequencing data, control, J20	Gene Expression Omnibus	GSE125957

**Experimental models: Organisms/strains**		

Drosophila: P{w[+mW.hs] = GawB}elav[C155]	Bloomington Drosophila Stock Center	458
Drosophila: y[1] sc[∗] v[1]; P{y[+t7.7] v[+t1.8] = TRiP.HMS00886}attP2	Bloomington Drosophila Stock Center	33,936
Drosophila: w[1118]; P{w[+mC]=UAS-Moe.T559D.MYC}2	Bloomington Drosophila Stock Center	8630
Drosophila: w[1118]	Bloomington Drosophila Stock Center	3605
Drosophila: *UAS-tau*^*R406W*^	Mel Feany	*UAS-tau*^*R406W*^

**Software and algorithms**		

Trimmomatic (v.0.36)	Bolger et al.^102^	N/A
FastQC	Bittencourt,^103^	N/A
Salmon (v.0.13.1)	Patro et al.^104^	N/A
DESeq2 (v1.24)	Love et al.^105^	N/A
WGCNA package	Langfelder and Horvath,^25^	N/A
clusterProfiler (v3.04)	Yu et al.^106^	N/A
DOSE (v3.11)	Yu et al.^107^	N/A

### Resource availability

#### Lead contact

Further information and requests for resources and reagents should be directed to and will be fulfilled by the lead contact Bess Frost (bfrost@uthscsa.edu).

#### Materials availability

This study did not generate new unique reagents.

### Experimental model and subject details

#### Drosophila

Crosses and aging were performed at 25°C with a 12 hour light/dark cycle at 60% relative humidity on a standard diet (Bloomington formulation). Panneuronal expression of transgenes, including RNAi-mediated knockdown, in *Drosophila* was achieved using the GAL4/UAS system with the *elav* promoter driving GAL4 expression.^108^ An equal number of males and females were used in all *Drosophila* assays. Full genotypes and sources are listed in Table S10.

#### Human tissue

Human brain tissue was obtained from the Mayo Clinic Brain Bank. Human subject information for Moesin staining is included in Table S9.

### Method details

#### RNA sequencing and differential gene expression analyses

##### Human

RNA-seq data was available for 76 patients with Alzheimer’s disease (42.1% male, 57.9% female) and 48 non-demented controls (52.1% males, 47.9% female). Additional information for each patient brain is provided in Table S1 and the Accelerating Medicines Partnership – Alzheimer’s disease (AMP-AD) Knowledge Portal (Synapse ID: syn3163039). Whole-transcriptome data was downloaded from the AMP-AD Knowledge Portal (Synapse ID: syn3163039). Gene expression data from temporal cortex was generated by the Mayo Clinic Brain Bank using Illumina HiSeq 2000-based next-generation 101 bp paired-end sequencing. FASTQ files were trimmed with Trimmomatic (v.0.36)^102^ to remove adapters and low-quality reads. FastQC^103^ was used to evaluate read quality before and after trimming. Trimmed FASTQ files were mapped and aligned to the *Homo sapiens* transcriptome (Gencode v31) using Salmon (v.0.13.1).^104^ Differential expression analysis was performed using DESeq2 (v1.24).^105^ Trimmomatic and Salmon tools were run using the resources provided by the University of Texas Health San Antonio Bioinformatics Core Facility. Genes with an adjusted p value of less than 0.05 were considered significant.

##### Mouse

RNA-seq data from rTg4510 and APP mice were obtained from the Gene Expression Omnibus (GEO) (rTg4510 GEO: GSE186140; J20 GEO: GSE125957).^109^ FASTQ files were downloaded from GEO and trimmed with Trimmomatic (v.0.36)^102^ to remove adapters and low-quality reads. FastQC^103^ was used to evaluate the quality of the reads before and after trimming. Trimmed FASTQ files were mapped and aligned to the *Mus musculus* transcriptome (Gencode M22) using Salmon (v.0.13.1).^104^ Differential expression analysis was performed using DESeq2 (v1.24).^105^ Mouse Trimmomatic and Salmon tools were run using resources on the TACC Lonestar5 cluster. Genes with an adjusted p value of less than 0.05 were considered significant.

#### Weighted gene co-expression network analyses (WGCNA)

Each of the networks in this study were constructed using the R WGCNA package and methodologies previously described.^25^ Here, gene expression data was normalized by transcripts per million (TPM) and log base two transformation. From more than 15,000 genes, 8,000 of the most varying genes were preliminarily selected for network construction. Genes were removed if they contained too many missing values (minimal fraction = 1/2) if mean expression was less than two TPM or if they had zero variance. Outlier samples were detected by hierarchical clustering using the R core Stats package. In order to obtain biologically meaningful networks and understand the directionality of node profiles, we constructed signed hybrid adjacency matrices where the absolute value of the Pearson correlation measures gene is the co-expression similarity, and a_ij_ represents the resulting adjacency that measures the connection strengths *a*_*ij*_ *= |cor(x*_*i*_*, x*_*j*_*)|* ^*ß*^. Network connectivity ${k_{i}=\sum }_{u\neq i}^{aiu}$ is defined as the sum of connection strengths with other genes. Soft-thresholding powers (ß) were selected using the scale-free criterion in which the network connectivity distribution of nodes approximately followed inverse power law *p(k)∼k*^*∼γ*^.^110^ Due to limitations in data visualization software, networks were further restricted to the 5,000 most connected genes. Modules were defined as genes with high topological overlap where the overlap between genes *i* and *j* was measured using $\omega =\frac{l_{ij}+a_{ij}}{\min\{k_{i},+1-a_{ij}}$. Modules were identified by average linking hierarchical clustering along with the distance calculated from the topological overlap matrix as a measure of dissimilarity $d_{ij}^{\omega }$ *= 1 - ω*_*ij*_. Module cut heights ranged from 0.1-0.25 based on the number of modules detected and cluster distancing. Only co-expressed genes in groups of 100 genes or more were considered modules. Hub genes for each module were identified by ranking genes according to their intramodular connectivity (k_in_) and selecting the top 1-5% of the most connected genes. In each case, modules were assessed for enrichment in biological processes using the enrichGO algorithm provided by clusterProfiler (v3.04).^106^ Associations with biological processes were considered significant if adjusted p values (false discovery rate) were less than 0.05. To identify gene-disease associations for each module, we utilized DOSE (v3.11)^107^ in conjunction with the enrichDGN algorithm. Gene-disease associations were considered significant if adjusted p values (false discovery rate) were less than 0.05.

##### Module preservation analysis

Module preservation analysis was performed using methodologies previously described.^40^ The gene clustering dendrogram of the tau mouse network was re-created using the same network construction techniques as in the human network. To restrict our analysis to the most preserved and connected genes, we only included genes with scaled connectivities greater than 0.1. Determination of preservation statistics was performed using the modulePreservation function from the WGCNA package and corrected for multiple testing using Bonferroni’s correction. The comprehensive set of module preservation statistics is provided in Figure S2. See Langfelder et al. for complete list of definitions and glossary.^40^

##### Principal component analyses

For multi-dimensional scaling plots depicted in Figures 1 and 2, module eigengene and whole-network matrices were analyzed using the prcomp function from the R core package Stats. Whole networks and module eigengenes from each of their respective networks were analyzed using the measure of dissimilarity previously calculated.

#### Immunofluorescence and histology

##### Human

For Moesin immunofluorescence, frozen pieces of brain tissue from temporal cortex were sectioned at -20 ˚C and transferred to microscope slides. Samples were then warmed to room temperature and immediately incubated in 4% PFA at room temperature for 10 minutes. Slides were then rinsed in diH_2_O and immersed in sodium citrate buffer (10 mM sodium citrate, 0.05% Tween 20, pH 6.0) and incubated above a 240 W LED light source (HTG Supply, Cat. No. LED-6B240) at 4°C for four hours to reduce lipofuscin autofluorescence. Next, slides were incubated in blocking solution (2% non-fat milk in PBS plus 0.3% TritonX (PBS_Tr_)) at 4°C for 30 minutes. Following non-specific blocking, slides were incubated overnight in blocking solution containing primary antibodies. The following day, slides were rinsed three times in PBS_Tr_ and incubated in blocking solution containing secondary antibodies at room temperature for one hour. Next slides were rinse three times using PBS_Tr_, mounted with DAPI containing media, and coverslipped. Brains were visualized by confocal microscopy (Zeiss LSM 780 NLO with Examiner, Zeiss LSM 810 with Airyscan), and ImageJ.^111^ Immunofluorescence was quantified by measuring average Moesin signal intensity within the nucleus of 50 neurons per biological replicate. For each sample, images were converted to 8-bit binary Z-projections using the Max Intensity projection setting and thresholded with the default parameters in ImageJ. Total fluorescence for each biological replicate was calculated by taking the product of the mean gray value and percent area for each of the 50 regions of interest selected and averaged. Antibodies, reagents, concentrations, and sources are listed in Table S11.

##### Drosophila

For α-catenin, shotgun, Nrg, and Fas2 immunofluorescence, *Drosophila* brains were dissected in PBS, fixed in methanol for 10 minutes and adhered to microscope slides. Slides were rinsed in diH20 and washed using PBS followed by blocking with 2% milk in PBS plus 0.3% PBS_Tr_ for 30 minutes. Slides were incubated with primary antibodies diluted in blocking solution overnight at 4°C. After incubation with primary antibodies, slides were washed with 0.3% PBS_Tr_ and incubated with Alexa 488-, Alexa555-, or Alexa647-conjugated secondary antibodies diluted in blocking solution for two hours at room temperature. Lastly, slides were washed again and incubated with DAPI for two minutes to stain nuclei before cover slipping.

For phalloidin staining, dissected *Drosophila* brains were fixed in 4% PFA for ten minutes and prepared for staining according to the manufacturer’s protocol (Cell Signaling Technology). Brains were visualized by confocal microscopy (Zeiss LSM 780 NLO with Examiner, Zeiss LSM 810 with Airyscan), and ImageJ^112^ was used for analysis. Total fluorescence was measured by taking Z-projections of stacked images of the entire *Drosophila* brain using the Max Intensity projection settings in ImageJ. For each biological replicate, the product of the mean gray value and percent area was calculated from thresholded 8-bit binary channels containing either α-catenin, shotgun, Nrg, and Fas2 using the default thresholding method in ImageJ. To quantify high signal foci from images of phalloidin staining, we utilized the Analyze Particles tool from ImageJ.^112^ Briefly, stacked images were converted to z-projections using MaxEntropy thresholding to exclude low signal and background. Z-projected images were then converted to 8-bit binary images using MaxEntropy thresholding. To reliably quantify the number of particles per brain we excluded particles outside of the brain with sizes less than 0.1 pixelˆ2 or greater than 100 pixelsˆ2. Circularity was left to the default setting.

Proliferating cell nuclear antigen (PCNA) analyses were performed using 4 μm sections from formalin-fixed, paraffin-embedded *Drosophila* heads. Sections were adhered to microscope slides then deparaffinized and dehydrated using a xylene and ethanol series of rinses and washes. To improve signal detection, slides were heated to 100°C for 15 minutes in 1 L of 10 mM sodium citrate in 0.05% Tween 20, pH 6.0. Slides were then washed in PBS and blocked using 2% milk in 0.3% PBS_Tr_ for 30 minutes. Next, slides were incubated with an anti-PCNA antibody diluted in blocking solution overnight at 4°C. Secondary detection was performed with a biotinylated secondary antibody and diaminobenzidine (DAB) according to the manufacturer’s protocol (Vector Laboratories). PCNA-positive foci were counted throughout the entire brain by brightfield microscopy (Nikon Eclipse Ci-L). Antibodies, reagents, concentrations, and sources are listed in Table S11.

##### TUNEL

To measure neuronal death in *Drosophila* brains, we used a commercially available DNA fragmentation detection kit for TUNEL staining (Calbiochem, TdT FragEL) using 4 μm sections of formalin-fixed, paraffin embedded *Drosophila* brain tissue. As directed in the provided protocol, DAB (Vector Laboratories, SK-4105) was used for detection of biotin-labelled deoxynucleotides at exposed ends of DNA fragments. Brightfield microscopy (Nikon Eclipse Ci-L) was used to quantify TUNEL-positive cells throughout the *Drosophila* brain.

#### Western blotting

Frozen *Drosophila* heads were homogenized in 15 μl of 2X Laemmli sample buffer, heated for 5 minutes at 95°C, and analyzed by 4–20% or 7.5% SDS–PAGE using the Bio-Rad mini-PROTEAN Tetra Cell. Polyacrylamide gels were transferred at 4°C for 90 minutes at 90 V to nitrocellulose or PVDF membranes using the Bio-Rad Mini Trans-Blot Cell and Towbin buffer.^113^ Equal loading was assessed by Ponceau S staining. Membranes were then incubated at 4°C for 30 minutes in a blocking solution made up of 2% milk in PBS plus 0.05% Tween (PBS_Tw_) followed by incubation with primary antibodies overnight at 4°C with gentle rocking. Membranes were then washed using 0.05% PBS_Tw_ and incubated with HRP-conjugated secondary antibodies for two hours at room temperature. Blots were developed with an enhanced chemiluminescent substrate and imaged using the ProteinSimple FluorChem HD2 system. Antibodies, reagents, concentrations, and sources are listed in Table S11.

### Quantification and statistical analysis

Every reported *n* is the number of biologically independent replicates. Except when noted otherwise, statistical analyses were performed using a one-way ANOVA with Tukey test when comparing among multiple genotypes and a two-tailed, unpaired Student’s t-test when comparing two genotypes. Data distribution was assumed to be normal, but this was not formally tested. For RNA-seq analysis, a two-sided Wald test was used to calculate false discovery rates (FDR-adjusted p value).^114^ A p value less than 0.05 was considered significant unless otherwise specified. Sample sizes are similar to or greater than those reported in previous publications. Samples were randomized in all *Drosophila* studies. Investigators were blinded, when possible, to genotype in all immunohistochemistry and immunofluorescence.

## Data Availability

This study did not generate new data or code.

## Supplemental information

Document S1. Figures S1–S5

Table S1. Human subject information for WGNCA, related to Figure 1

Table S2. Differential expression analysis of genes within each module of the human WGCNA, related to Figure 1

Table S3. Gene Ontology analysis of modules within the human WGCNA, related to Figure 1

Table S4. DisGeNET analysis of modules within the human WGCNA, related to Figure 1

Table S5. Gene Ontology analysis of modules within the tau transgenic WGCNA, related to Figure 2

Table S6. Gene Ontology analysis of modules within the APP transgenic WGCNA, related to Figure 2

Table S7. APP transgenic WGCNA, related to Figure 2

Table S8. Tau transgenic WGCNA, related to Figure 2

Table S9. Human subject information for Moesin staining, related to Figure 3

Table S10. Full genotypes and sources of Drosophila stocks and crosses, related to Figures 3–5

Table S11. Antibody and cell staining sources and dilution information, related to Figures 3–5
