# Supplementary material for: Moesin is an effector of tau-induced actin overstabilization, cell cycle activation, and neurotoxicity in Alzheimer’s disease
Source: iScience. 2023 Feb 8;26(3):106152. doi: 10.1016/j.isci.2023.106152 (PMC9984563; doi:10.1016/j.isci.2023.106152)
Supplement: Document S1. Figures S1–S5 [file mmc1.pdf]

iScience, Volume *26*

Supplemental information

Moesin is an effector of tau-induced actin overstabilization, cell cycle activation, and neurotoxicity in Alzheimer's disease

Adrian Beckmann, Paulino Ramirez, Maria Gamez, Elias Gonzalez, Jasmine De Mange, Kevin F. Bieniek, William J. Ray, and Bess Frost

SUPPLEMENTAL INFORMATION

Supplemental Figures

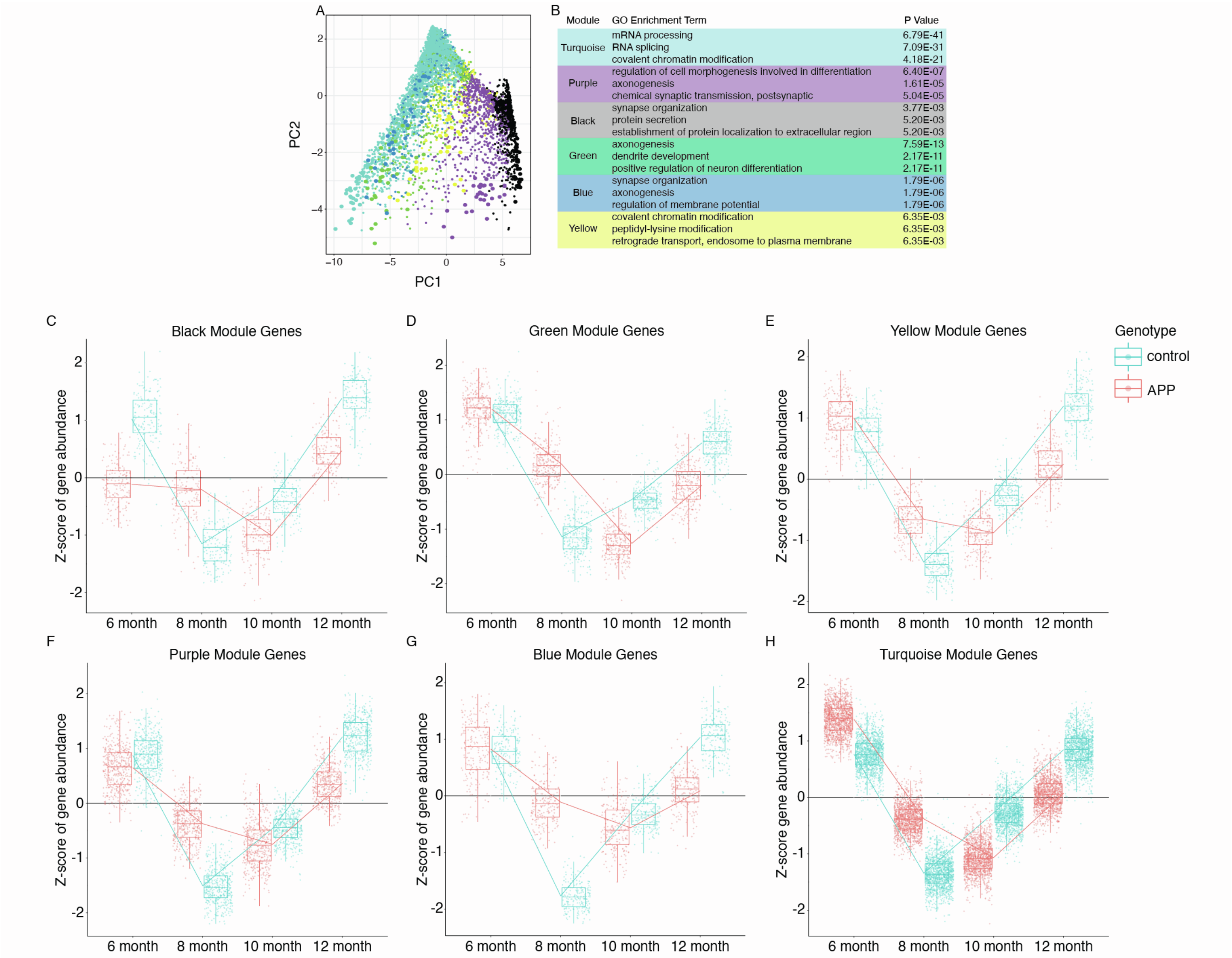

Module	GO Enrichment Term	P Value
Turquoise	mRNA processing	6.79E-41
	RNA splicing	7.09E-31
	covalent chromatin modification	4.18E-21
Purple	regulation of cell morphogenesis involved in differentiation	6.40E-07
	axonogenesis	1.61E-05
	chemical synaptic transmission, postsynaptic	5.04E-05
Black	synapse organization	3.77E-03
	protein secretion	5.20E-03
	establishment of protein localization to extracellular region	5.20E-03
Green	axonogenesis	7.59E-13
	dendrite development	2.17E-11
	positive regulation of neuron differentiation	2.17E-11
Blue	synapse organization	1.79E-06
	axonogenesis	1.79E-06
	regulation of membrane potential	1.79E-06
Yellow	covalent chromatin modification	6.35E-03
	peptidyl-lysine modification	6.35E-03
	retrograde transport, endosome to plasma membrane	6.35E-03

Figure S1. Modules in the APP transgenic network are enriched for biological processes associated with neurotransmission but do not significantly change across disease staging, Related to Figure 2. Network analysis from APP transgenic mouse hippocampi show module directionality and magnitude change across aging. **(A)** Multidimensional scaling plot of the entire APP transgenic network using principal component two as a function of principal component one. Each point is a single gene. **(B)** Biological processes with the highest degree of significant enrichment based on Gene Ontology. Tables containing enriched biological processes and module information are provided in Supplementary Tables 6 and 7. Box and whisker plots show gene expression changes from the **(C)** black, **(D)** green, **(E)** yellow, **(F)** purple, **(G)** blue, and **(H)** turquoise modules at six, eight, ten, and twelve months of age.

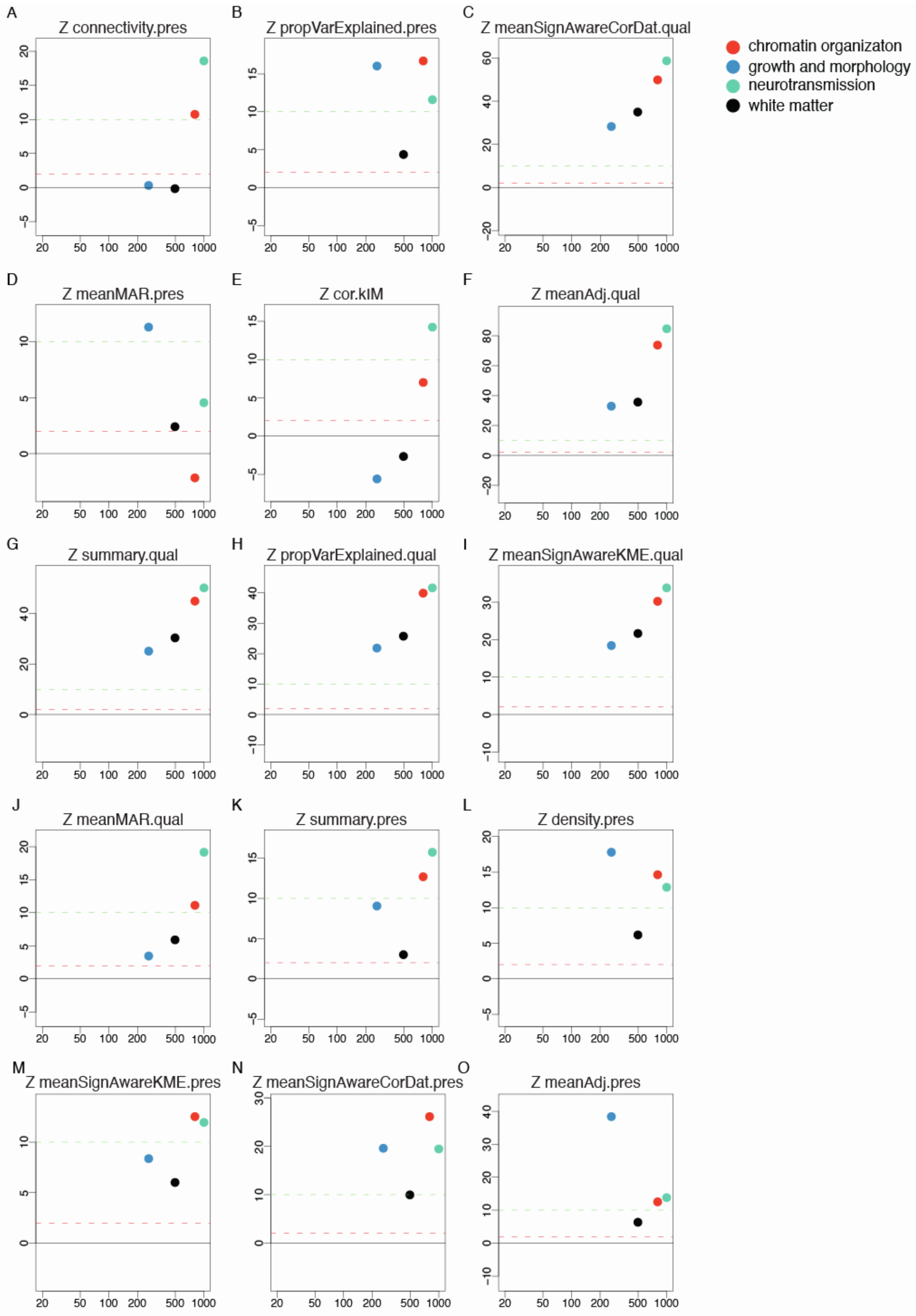

Figure S2. Different measures of the human network structure are preserved in the tau transgenic network, Related to Figure 2. (**A-O**) Preservation statistics calculated for the preservation of the human network (reference) in the mouse (test) network. Each preservation statistic measures a different aspect of the human network preservation and is reported using Z statistics scores. For each graph, module size is labeled along the x-axis and Z scores on the y-axis. Thresholds reflect scoring in which Zscores > 10 are evidence of strong preservation, Zscores > 2 and < 10 are evidence of weak to moderate preservation, and Zscores < 2 are evidence of no module preservation. See Langfelder *et al*. for complete list of definitions and glossary (Langfelder *et al*., 2011).

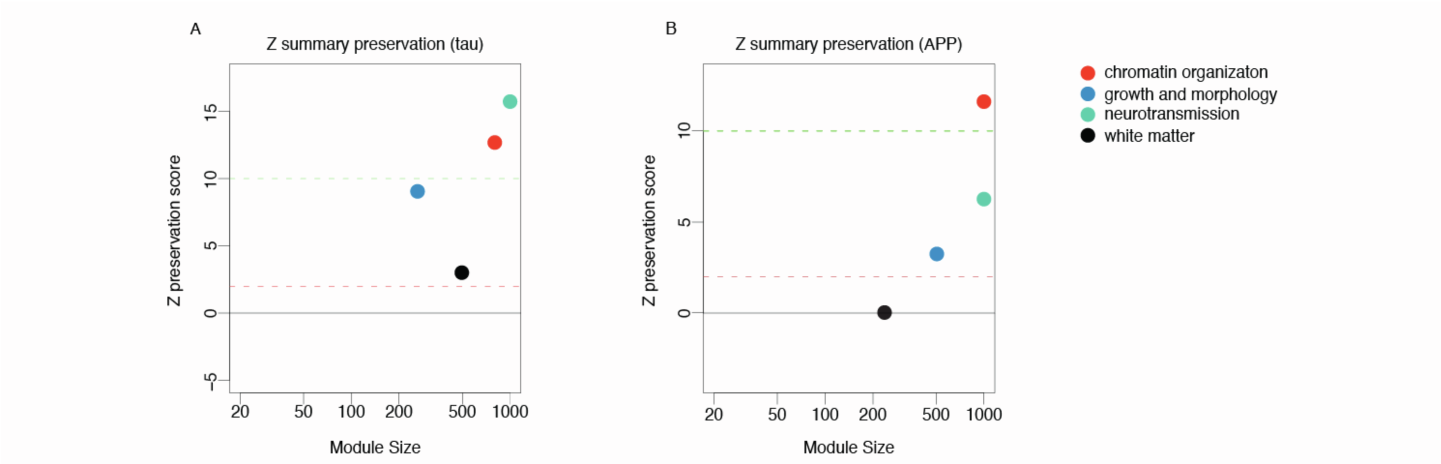

Figure S3. Turquoise, blue, and red modules of the human network are preserved in the mouse network, Related to Figure 2. Summary statistics for human module preservation in the **(A)** tau and **(B)** APP transgenic mouse networks. Composite of all preservation statistics calculated from module preservation including using summarized statistics from Zdensity and Zconnectivity-based statistics. Thresholds reflect scoring which dictates that Zscores > 10 are evidence of strong preservation, Zscores > 2 and < 10 are evidence of weak to moderate preservation, and Zscores < 2 are no evidence of module preservation. Z-scores are calculated by: -$\log_{10}$(p-value). See Langfelder *et al.* for complete list of definitions and glossary(Langfelder *et al.*, 2011).

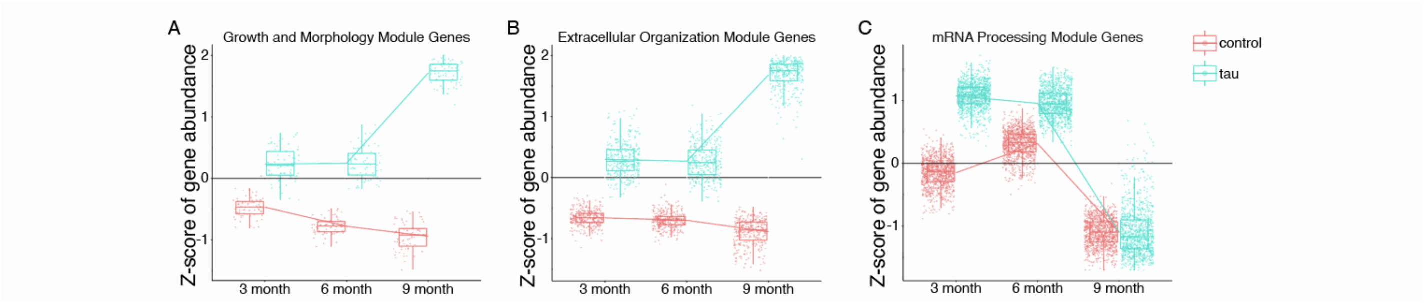

Figure S4. Differential expression of modules in control versus tau transgenic mouse brains across disease stage, Related to Figure 2. Box and whisker plots show gene expression changes from the **(A)** yellow, **(B)** green and **(C)** red modules at three, six, and nine months of age.

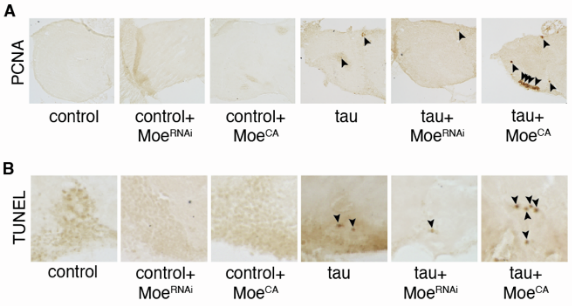

Figure S5. Moesin activation facilitates cell cycle activation and consequent neuronal death in tauopathy, Related to Figure 5. Representative brightfield microscopy images of Moesin activation and Moesin knockdown based on (**A**) PCNA and (**B**) TUNEL staining in control and tau transgenic *Drosophila*. All flies are ten days old. Full genotypes are listed in **Table S10**.

Supplemental Tables

Table S1. Human subject information for WGNCA, Related to Figure 1.
Table S2. Differential expression analysis of genes within each module of the human WGCNA, Related to Figure 1.
Table S3. Gene Ontology analysis of modules within the human WGCNA, Related to Figure 1.
Table S4. DisGeNET analysis of modules within the human WGCNA, Related to Figure 1.
Table S5. Gene Ontology analysis of modules within the tau transgenic WGCNA, Related to Figure 2.
Table S6. Gene Ontology analysis of modules within the APP transgenic WGCNA, Related to Figure 2.
Table S7. APP transgenic WGCNA, Related to Figure 2.
Table S8. Tau transgenic WGCNA, Related to Figure 2.
Table S9. Human subject information for Moesin staining, Related to Figure 3.
Table S10. Full genotypes and sources of *Drosophila* stocks and crosses, Related to Figures 3-5.
Table S11. Antibody and cell staining sources and dilution information, Related to Figures 3-5.
